# Microglial inflammation and phagocytosis in Alzheimer's disease: Potential therapeutic targets

**DOI:** 10.1111/bph.14618

**Published:** 2019-05-11

**Authors:** Sohaib Nizami, Hazel Hall‐Roberts, Sharat Warrier, Sally A. Cowley, Elena Di Daniel

**Affiliations:** ^1^ Alzheimer's Research UK Oxford Drug Discovery Institute, Nuffield Department of Medicine University of Oxford Oxford UK; ^2^ James Martin Stem Cell Facility, Sir William Dunn School of Pathology University of Oxford Oxford UK

## Abstract

One of the largest unmet medical needs is a disease‐modifying treatment for Alzheimer's disease (AD). Recently, the role of microglia in disease, particularly AD, has gained great interest, following the identification of several disease risk‐associated genes that are highly expressed in microglia. Microglia play a critical homeostatic role in the brain, with neuroinflammatory and phagocytic mechanisms being of particular importance. Here, we review the role of NLRP3, the complement system, and the triggering receptor expressed in myeloid cells 2 (TREM2) in modulating microglial functions. We have reviewed the targets, their molecular pathways and the therapeutic interventions aimed at modulating these targets, in the hope of discovering a novel therapeutic approach for the treatment of AD.

**Linked Articles:**

This article is part of a themed section on Therapeutics for Dementia and Alzheimer's Disease: New Directions for Precision Medicine. To view the other articles in this section visit http://onlinelibrary.wiley.com/doi/10.1111/bph.v176.18/issuetoc

**Abbreviations**
Aβ: β‐amyloid
AD: Alzheimer's disease
ADAM: a disintegrin and metalloproteinase
APP: amyloid precursor protein
ASC: apoptosis‐associated speck‐like protein containing a caspase recruitment domain
BBB: blood–brain barrier
CAPS: cryopyrin‐associated periodic syndromes
CR1: complement receptor 1
DAF: decay accelerating factor
DAMPs: damage‐associated molecular patterns
DAP12: DNAX‐activation protein 12
GWAS: genome‐wide association studies
KO: knockout
LOAD: late‐onset Alzheimer's disease
MAC: membrane attack complex
NLRP3: NOD‐like receptor family pyrin domain containing 3
PAMPs: pathogen‐associated molecular patterns
PS1: presenilin‐1
SHIP‐1: SH‐2 containing inositol 5′ polyphosphatase‐1
SNP: single nucleotide polymorphism
sTREM2: soluble triggering receptor expressed in myeloid cells 2
TREM2: triggering receptor expressed in myeloid cells 2

## 1 INTRODUCTION

Alzheimer's disease (AD) is an age‐related neurodegenerative disorder and the most common cause of dementia. In the United States alone, it is estimated that 5.5 million adults have AD, and by 2050, it is expected to affect 13.8 million adults (Taylor, Greenlund, McGuire, Lu, & Croft, 2017). Given that all patients at the late stage of the disease require full‐time care, this puts an immense financial burden on the state and is reflected in the global cost of dementia, estimated to be $818 billion in 2015 (Wimo et al., 2017). Patients develop the disease over years or perhaps decades, and it classically presents with memory loss and a diverse spectrum of additional symptoms. The diagnosis is confirmed by characteristic neuropathologies: brain amyloid plaques and neurofibrillary tangles, microgliosis, astrogliosis, dystrophic neurites, and progressive cerebral atrophy (Hansen, Hanson, & Sheng, 2018; Herrup, 2015). Neuronal loss can start years before symptoms develop and initially localises to the hippocampus and entorhinal cortex in early stages of disease. The most widely accepted explanation for AD, known as the “amyloid hypothesis,” proposes that misfolding and aggregation of the peptide β‐amyloid (Aβ) causes a linear cascade of pathology that results in both extracellular amyloid plaques and intracellular deposition of misfolded Tau protein that forms neurofibrillary tangles (Chen et al., 2017; C. C. Tan, Zhang, Tan, & Yu, 2018). Since the amyloid hypothesis was first proposed, the linearity of this mechanism has been brought into question, with suggestions that amyloid and Tau pathologies may occur concurrently or even independently (De Strooper & Karran, 2016). Some have gone further, to suggest that there are several independent causes of AD, which include neuroinflammation, calcium dysregulation, mitochondrial dysfunction, and impairment of the autophagy‐lysosome degradation pathway (Herrup, 2015).

One of the exciting developments in the field has been the identification of genetic risk factors for AD, which allows for the understanding of root causes or risks and can be used for target validation for AD. Historically, the role of familial genes such as those for amyloid precursor protein (APP) and presenilin‐1 (PS1) has supported the amyloid hypothesis (Herrup, 2015). More recently, genome‐wide association studies (GWAS) have highlighted that several microglia‐specific genes are significantly associated with AD risk (Villegas‐Llerena, Phillips, Garcia‐Reitboeck, Hardy, & Pocock, 2016). This has moved the attention of the field towards the physiological and pathophysiological role of microglia, which are specialised brain tissue‐resident macrophages. Physiologically, microglia have a surveillance function, where they extend long branched processes to sample their micro‐environment and monitor the health of surrounding neurons and glia. Microglia also perform phagocytosis of debris and pathogens, regulate brain development, and mediate inflammatory responses to injury and infection. Microglia are understood to occupy several discrete functional states, traditionally termed “M1,” “M2,” and “M0” that correspond to pro‐inflammatory, anti‐inflammatory, or surveillance activities, respectively. This view of functional states is evolving, and microglia associated with AD lesions do not fit neatly into the traditional M1 or M2 classification (Du et al., 2017). In the AD environment, the role of microglia may be both neuroprotective and neurotoxic, the balance between which may change over time in a single individual (Du et al., 2017; Labzin, Heneka, & Latz, 2018). Reactive microglia release cytokines in the parenchyma, referred to as neuroinflammation, which can be beneficial if the response clears the site of injury or infection but detrimental if this reaction further damages the surrounding tissue (Ransohoff, 2016). Reduced phagocytic clearance of dying neurons and protein aggregates by microglia may allow waste to accumulate, resulting in microglial pro‐inflammatory activation in response to amyloid. On the other hand, inappropriate phagocytosis of synapses in AD has been postulated, based on evidence of complement‐mediated synaptic pruning in AD mouse models (Salter & Stevens, 2017; Vilalta & Brown, 2017). The aim of this review is to examine the function of these processes through targets that have been associated with AD and that are of growing interest in the field. The roles of the NOD‐like receptor family pyrin domain containing 3 (NLRP3) inflammasome in neuroinflammation (Guo, Callaway, & Ting, 2015), the complement system (Morgan, 2018), and the triggering receptor expressed in myeloid cells 2 (TREM2) in phagocytosis (Jay, von Saucken, & Landreth, 2017) are reviewed.

## 2 NLRP3 INFLAMMASOME

Neuroinflammation can involve numerous pathways and cytokines, and it is likely that several of them play an important role in AD pathology (F. Zhang & Jiang, 2015). Recently, focus has turned to the NLRP3 inflammasome, which, upon activation, triggers the cleavage of pro‐IL‐1β and pro‐IL‐18 to their active forms (Guo et al., 2015). In this section, we describe the NLR family, the NLRP3 inflammasome activation pathway, evidence that NLRP3 inflammasome activity may worsen AD pathology, and progress made to develop therapeutic agents targeted at reducing activation of the NLRP3 inflammasome.

### 2.1 The inflammasome family

The NLR family contains a carboxy‐terminal leucine‐rich repeat domain, a conserved central NACHT domain, and a variable amino‐terminal domain. This family includes NLRP1, NLRP2, NLRP3, NLRP6, NLRP7, NLRP12, and NLRC4 (Walsh, Muruve, & Power, 2014). NLRP1, NLRP2, and NLRP3 are the best characterised inflammasomes of the NLR family. NLRP3 is highly abundant in microglia compared to NLRP1 and NLRP2, which have a higher expression in neurons and astrocytes, respectively (de Rivero Vaccari, Dietrich, & Keane, 2014). It is largely for this reason that NLRP3 has attracted the most attention compared to other inflammasomes. It is also an attractive target because NLRP3 is downstream of converging signalling pathways that are stimulated by damage‐associated molecular patterns (DAMPs), motifs associated with damaged human cells such as HMGB1. In addition, it is also stimulated by pathogen‐associated molecular patterns (PAMPs), the biochemical motifs on invading pathogens, such as bacterial LPSs (Guo et al., 2015).

### 2.2 NLRP3 inflammasome activation pathway

**Figure 1:**
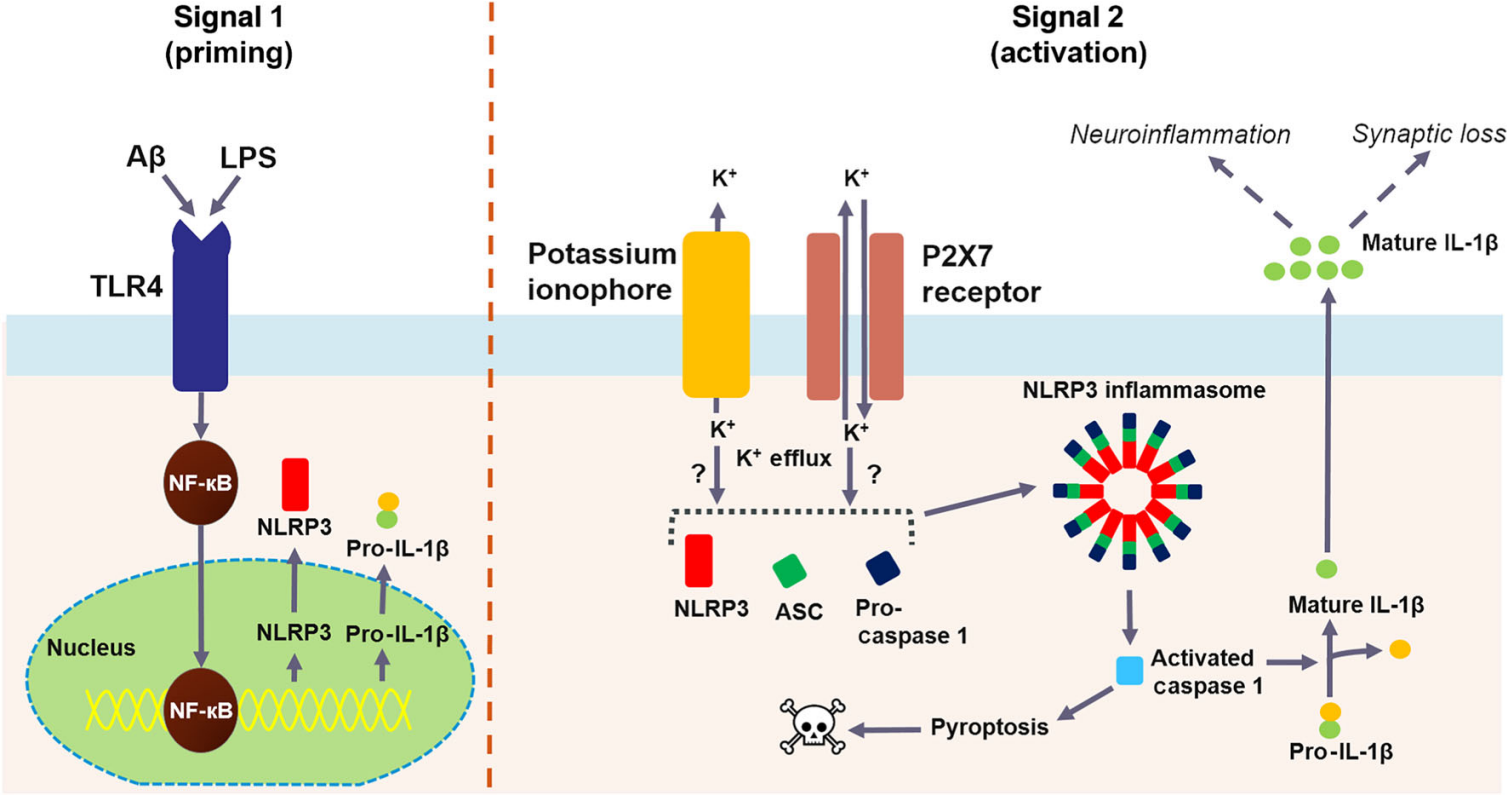
NLRP3 inflammasome activation pathway. Signal 1 (priming) involves the activation of the TLR4 receptors found on microglia through numerous stimuli that include Aβ_1–42_ and LPS. This results in the translocation of NF‐кB from the cytosol to the nucleus, which increases the transcription of NLRP3 and pro‐IL‐β. Following “priming,” the microglia need an additional stimulation from Signal 2 (activation). In this diagram, the efflux of K^+^ triggers the activation of the cascade that brings the NLRP3, ASC, and pro‐caspase 1 proteins together to form the NLRP3 inflammasome. This activates caspase 1, which cleaves IL‐1β and triggers pyroptosis resulting in cell death. The mature IL‐1β when released onto the surrounding cells exacerbates neuroinflammation and synaptic loss

Understanding the signalling pathways that activate the NLRP3 inflammasome is key to finding therapeutic intervention points. However, the NLRP3 inflammasome activation pathway has not yet been fully characterised. The role that calcium signalling, mitochondrial dysfunction, and destabilised lysosomes play in NLRP3 activation has not been fully elucidated (Zhou, Shi, Wang, Chen, & Zhang, 2016). There is a consensus that the canonical activation of the NLRP3 inflammasome requires two signals called “priming” (Signal 1) and “activation” (Signal 2) (Prochnicki, Mangan, & Latz, 2016). Signal 1 is initiated by PAMP‐ and DAMP‐mediated stimulation of toll‐like receptors, resulting in NF‐κB activation and up‐regulation of NLRP3, pro‐IL‐1β, and pro‐IL‐18 (Place & Kanneganti, 2018). Signal 2 is induced by a wide variety of stimuli, including ATP, viral RNA, and particulate matter (Guo et al., 2015). Signal 2 stimuli could trigger potassium efflux, which is required for the NLRP3 inflammasome complex formation, although the mechanistic link is poorly understood (He, Hara, & Nunez, 2016). The inflammasome complex is formed by oligomerisation of NLRP3 monomers, which recruit apoptosis‐associated speck‐like protein containing a caspase recruitment domain (ASC) via pyrin domain interactions. ASC recruits pro‐caspase‐1 via a caspase recruitment domain, triggering caspase‐1 cleavage and maturation. Active caspase‐1 cleaves pro‐IL‐1β and pro‐IL‐18 to mature IL‐1β and IL‐18, respectively, and these cytokines are released into the extracellular space by a nonconventional secretion pathway (de Rivero Vaccari et al., 2014; Walsh et al., 2014). Under certain conditions, an inflammatory form of cell death called pyroptosis can also be triggered by activated caspase‐1 through cleavage of gasdermin D (Jo, Kim, Shin, & Sasakawa, 2016; Malik & Kanneganti, 2017). See Figure 1.

### 2.3 NLRP3 inflammasome activation is a pathophysiological pathway in AD

Patients that have gain‐of‐function mutations in NLRP3 develop systemic auto‐inflammatory syndromes called cryopyrin‐associated periodic syndromes (CAPS; Finetti, Omenetti, Federici, Caorsi, & Gattorno, 2016). The NLRP3 gain‐of‐function mutations result in the spontaneous NLRP3 inflammasome formation without the addition of Signal 2. This results in unrestrained IL‐1β secretion that disrupts the homeostasis of surrounding cells. Mice that have NLRP3 gain‐of‐function‐associated mutations display systemic inflammation and poor growth similar to CAPS patients (Bonar et al., 2012). Treatment with rilonacept, canakinumab or anakinra, compounds that act to suppress IL‐1β have greatly improved the life expectancy of CAPS patients, indicating that uncontrolled inflammation is damaging. Interestingly, patients with the most severe form of CAPS also display disruption of mental development (Finetti et al., 2016). This suggests that excessive IL‐1β production is detrimental to neuronal development and impairs health.

There is growing evidence to support the hypothesis that NLRP3 might be a crucial therapeutic target as it is chronically active in AD. A systematic study of immune system gene expression in healthy aging and in AD cases, in a region‐ and gender‐specific manner, concluded that there is a critical involvement of inflammation in the preclinical stage of AD with both caspase‐1 and IL‐1β up‐regulated (Cribbs et al., 2012). Histological evidence supports these findings and shows elevated caspase‐1 and ASC specks within microglia in brain sections of patients with AD (Heneka et al., 2013; Venegas et al., 2017). It may be reasonable to suspect genetic changes to support the histological data, and in fact, the rs2027432 NLRP3 polymorphism is associated with late‐onset Alzheimer's disease (LOAD) risk in a study that included 2,292 Han Chinese subjects (M. S. Tan et al., 2013). The same single nucleotide polymorphism (SNP) increased IL‐1β production through the up‐regulation of NLRP3 mRNA expression (A. Q. Zhang et al., 2011). Therefore, this SNP could potentially increase the transcriptional activity of the NLRP3 promoter resulting in an increase in NLRP3 expression in AD patients. These observations need to be confirmed in other cohorts in order to demonstrate the relevance to a broader AD population. However, there is a lack of evidence from GWAS that NLRP3 or the other components of the inflammasome are risk factors (Villegas‐Llerena et al., 2016). This may suggest that other genetic risk factors may modulate the NLRP3 inflammasome pathway, excessively stimulating IL‐1β production.

Evidence from rodent AD models indicates that genetic or pharmacological inhibition of the NLRP3 inflammasome pathway is neuroprotective. Inhibition of NLRP3 with low MW inhibitors, or knockout (KO) of NLRP3 expression, reduced Aβ burden and improved memory in plaque‐containing mice overexpressing human APP/PS1 mutations (Dempsey et al., 2017; Heneka et al., 2013). IL‐1β production is elevated in APP/PS1 mice relative to wild‐type mice and is significantly attenuated by NLRP3 KO. Furthermore, APP/PS1 mice exhibit synaptic deficits and an impairment of spatial learning memory that is fully reversed by NLRP3 KO. Amyloid deposition is also significantly reduced in APP/PS1‐NLRP3 KO mice, compared to APP/PS1 mice (Heneka et al., 2013). The possible explanation for this is that the NLRP3 deficiency improves microglial‐mediated phagocytosis of Aβ. In addition, when ASC KO mice were crossed with APP/PS1 transgenic mice, there was a significant reduction of Aβ in mice aged 8–12 months. One hypothesis is that ASC specks are released into the extracellular space upon microglial NLRP3 activation, which nucleate Aβ aggregation, contributing to the spread of Aβ pathology (Venegas et al., 2017).

Inhibition of the NLRP3 inflammasome activation pathway would prevent excessive IL‐1β production, thereby limiting damage to surrounding tissue. Numerous mechanisms suggest that IL‐1β is damaging in AD. IL‐1β enhances the production of S100β (Sheng et al., 1996), a protein associated with dementia in the elderly (Lambert et al., 2007). S100β is normally localised to the cytoplasm in astrocytes, so its presence in serum may indicate astrocyte dysfunction or damage (Shiotani et al., 2004). Aβ can activate the NLRP3 inflammasome and increase production of IL‐1β. Fibrillary Aβ_1–42_ can act as a priming agent for Signal 1 through the up‐regulation of IL‐1β mRNA expression (Stewart et al., 2010). Microglia that internalise Aβ_1–42_, in vitro and in vivo, show destabilised lysosomes, triggering the release of cathepsin B that activates the NLRP3 inflammasome pathway (Halle et al., 2008). IL‐1β production from microglia exacerbates Tau pathology in neurons in a glial‐neuronal coculture model (Li, Liu, Barger, & Griffin, 2003). Activated microglia released IL‐1β, resulting in Tau hyperphosphorylation and reduction in synaptophysin levels in neurons, therefore indicating neuronal toxicity. MAPK p38, which is implicated in Tau phosphorylation, is up‐regulated by IL‐1β in vivo (Kitazawa et al., 2011). This suggests a possible mechanism through which IL‐1β promotes the formation of neurofibrillary Tau protein tangles (Barron, Gartlon, Dawson, Atkinson, & Pardon, 2017).

There have been conflicting studies that examined whether IL‐1β is beneficial or detrimental in neurodegeneration. Sustained IL‐1β expression in an IL‐1β transgenic mouse model resulted in leukocyte infiltration to the brain, but this did not result in neurodegeneration (Shaftel et al., 2007). Overexpression of IL‐1β promotes the non‐amyloidogenic APP processing pathway, resulting in the reduction of Aβ production (Kong et al., 2009; Tachida et al., 2008). In an APP/PS1 mouse model of AD, IL‐1β overexpression resulted in chronic neuroinflammatory response that ameliorated Aβ plaque pathology (Shaftel et al., 2007). A study found that microglia in AD brain from Braak V–VI subjects have reduced response to Aβ compared to APP/PS1 mice (Gutierrez & Vitorica, 2018).

The studies reviewed above suggest that increasing the release of pro‐inflammatory IL‐1β from microglia would reduce AD pathology. However, as in other diseases, uncontrolled inflammation is more likely to be deleterious to the surrounding tissue; therefore, it might be therapeutically beneficial to dampen the signal. In summary, CAPS patients associated with NLRP3 mutations have exacerbated IL‐1β production, which in the most severe cases impairs neuronal health. In vitro and in vivo studies are largely supportive of the hypothesis that excessive production of IL‐1β damages surrounding healthy neurons, and there is growing evidence that these mechanisms play a role in AD.

### 2.4 Development of small molecule inhibitors of the NLRP3 inflammasome pathway

There is interest in developing inhibitors for the NLRP3 inflammasome pathway. This is because of the evidence supporting NLRP3 inflammasome activation being detrimental in several diseases that currently have no adequate therapies. In addition, there are likely several points of the cascade that can be targeted to inhibit the NLRP3 inflammasome activation. Rilonacept, canakinumab, and anakinra are biological inhibitors of IL‐1β that are in use clinically. These biological inhibitors have limited ability to pass the blood–brain barrier (BBB) and have no effect on IL‐1β production or release. In addition, IL‐1β is downstream of several inflammasomes and, hence, we could expect side effects by blocking IL‐1β. Targeting upstream in the NLRP3 inflammasome pathway would be preferable to limit side effects. Low MW compounds that are inhibitors of the NLRP3 inflammasome pathway have been described. However, the molecular target has not been identified, which makes compound optimisation difficult.

MCC950, also known as CRID3, is a potent and selective inhibitor of NLRP3 inflammasome, in vitro and in vivo (Coll et al., 2015). It inhibits activation of the NLRP3 inflammasome by several stimuli, including ATP, nigericin, and silica. MCC950 is highly specific for the NLRP3 inflammasome over other inflammasomes and does not inhibit TLR‐mediated pathways. MCC950 was in a phase II clinical trial for rheumatoid arthritis, but the trial was stopped due to signs of liver toxicity (Mangan et al., 2018).

The fenamate class of non‐steroidal anti‐inflammatory drugs includes mefenamic acid and flufenamic acid. This class of compounds inhibits ATP‐ and nigericin‐mediated activation of the NLRP3 inflammasome (Daniels et al., 2016). This mechanism of action is independent of COX enzyme inhibition and seems to involve inhibition of the volume‐regulated anion channels (Swanton et al., 2018). Mefenamic acid and flufenamic acid have weak potency but are specific for NLRP3 over other inflammasomes. In addition, fenamate compounds have been shown to be neuroprotective in rodent models of AD (Daniels et al., 2016).

Complex morphological states of microglia make it difficult to develop treatments for neuroinflammation. For example, P2X7 receptors have an established pro‐inflammatory role, and therefore, potential therapies would be expected to inhibit the receptor. However, the receptors are also associated with efferocytosis, and this would suggest that P2X7 receptors should be stimulated (Sanz et al., 2009). Nevertheless, inhibitors of P2X7 receptors could also dampen NLRP3 inflammasome activation, as they prevent the efflux of potassium from the cell. P2X7 KO mice showed a reduction in the release of mature IL‐1β (Solle et al., 2001). AZD9056 and GSK1482160 are P2X7 receptor antagonists that have entered clinical trials for rheumatoid arthritis but were found not to be effective in inhibiting inflammation. There could be several reasons for this, for example, the presence of multiple isoforms of the protein (Di Virgilio, Dal Ben, Sarti, Giuliani, & Falzoni, 2017). In addition, the inhibition of P2X7 receptor achieved in vivo was not measured in the rheumatoid disease study.

In summary, NLRP3 is part of a large family that functions in response to PAMPs and DAMPs stimuli. It is a convergent point of various pathways that ultimately results in the release of pro‐inflammatory cytokines. There is increasing evidence that uncontrolled inflammation can occur through NLRP3 inflammasome activation. MCC950 is a potent and NLRP3‐selective low MW inhibitor, which demonstrates that it is possible to target the pathway pharmacologically. Yet, currently, no compound has successfully passed clinical trials, suggesting that there is an enormous unmet potential. Future work should focus on identifying NLRP3‐selective inhibitors with good safety profiles, to progress to clinical trials for AD.

## 3 COMPLEMENT

Complement is an ancient and powerful arm of the innate immune system, participating in the recognition, trafficking, elimination of pathogens and unwanted host material, and maintaining homeostasis. The high complexity of the system renders it prone to error (Ricklin, Reis, & Lambris, 2016). Gain‐ or loss‐of‐function mutations and other genetic abnormalities can dysregulate complement‐mediated signalling, leading to increased tissue damage and inflammation. Our understanding of complement function in the brain is limited, compared with other tissues. In this section, we will briefly describe the complement cascade, its role in the CNS, its putative involvement in AD pathogenesis, and potential therapeutic intervention points.

### 3.1 The key components of complement cascade

The complement cascade functions through the activation of either of three primary pathways: the classical pathway, the alternative pathway, and the lectin pathway (Morgan, 2018).

In brief, activation of the complement cascade leads to formation of the C3 convertase, the first complex common to all routes of activation. The binding of an antibody to an antigen activates the classical complement pathway, when the Fc region of an IgG or IgM binds to C1q. This cleaves the C1 complex to C1q, C1r, and C1s. C1s cleaves C4 to C4a and C4b and C2 to C2a and C2b. C4b associates with C2b to form the C3 convertase (C4bC2b), which then cleaves C3. In the alternative pathway, C3 is hydrolysed or C3b binds to a pathogen. Factor D cleaves Factor B to Bb. Bb can then bind to C3b to generate the C3 convertase (C3bBb). In the lectin pathway, mannose‐binding lectin‐associated serine proteases cleave C4 and C2, leading to the generation of the C3 convertase.

Once the C3 convertase has been assembled, it cleaves C3 protein into two important inflammatory mediators: C3a and C3b. The C3 convertase can also create a C5 convertase complex, which cleaves C5, forming two inflammatory mediators: C5a and C5b. The products of C3 and C5 cleavage have a variety of functions. C3a and C5a are peptide mediators of inflammation, initiating the production of chemokines, cytokines, and ROS. C3a and C5a are also chemotactic factors that recruit inflammatory cells to the site of insult. C3b directly binds to immune complexes, enabling opsonised particles to be recognised for phagocytosis by complement receptors. C5b initiates the formation of the terminal membrane attack complex (MAC/C5b‐9).

**Figure 2:**
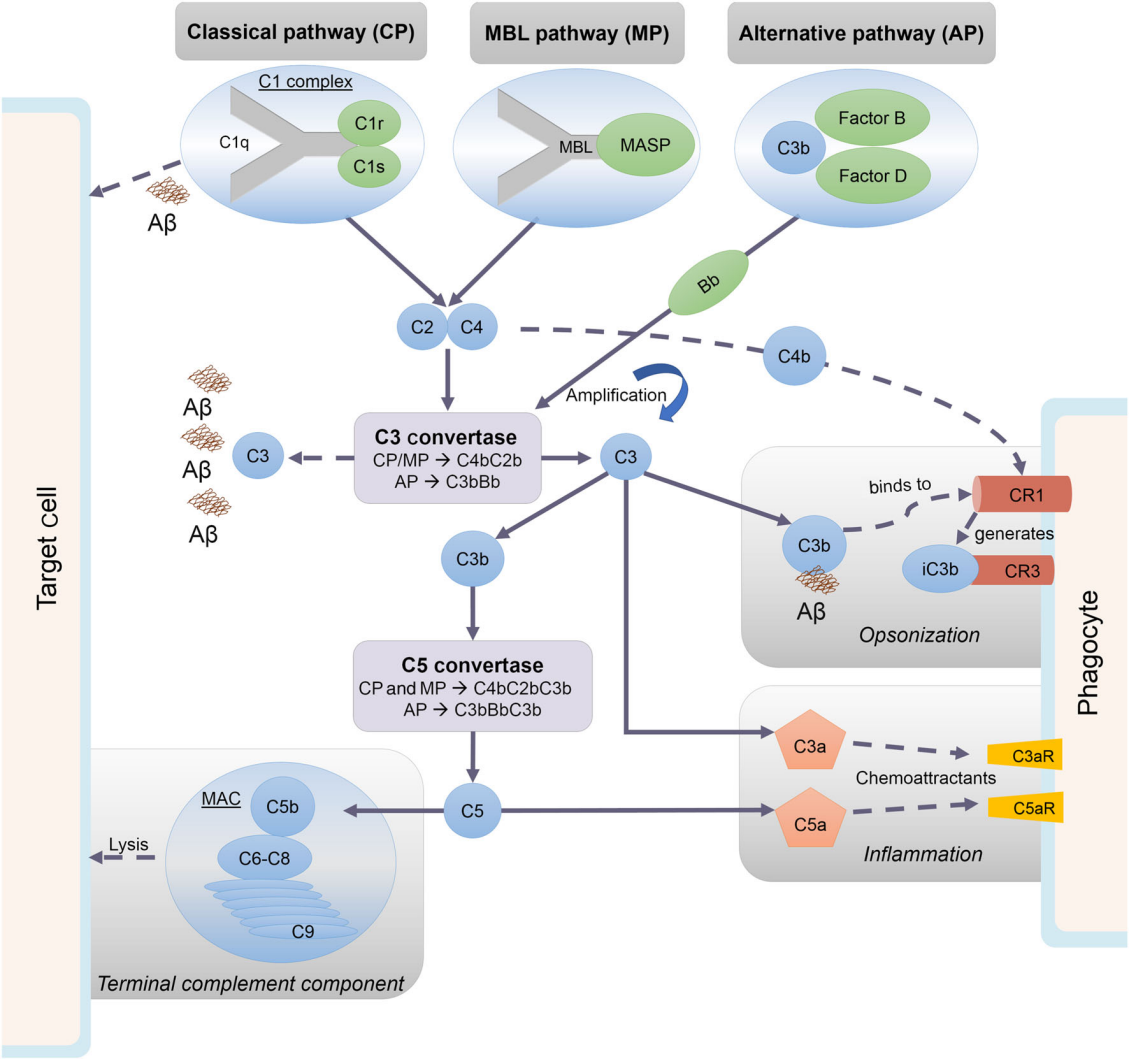
Complement activation pathways. The C3 convertase complexes C4bC2b or C3bBb are generated via three routes of activation: the classical pathway, the mannose‐binding lectin (MBL) pathway, and the alternative pathway. C3 convertase cleaves C3, to form C3a and C3b. In addition to a direct involvement of C3b and C3a in opsonisation and inflammation, respectively, C3b also forms part of the C5 convertase complex, which cleaves C5 and promotes assembly of the membrane attack complex (MAC)

The classical and alternative pathways ensure the cyclic low‐level background activity of the complement system. To tightly regulate the system, various checkpoints are in place, such as a decay accelerating factor (DAF), cleavage of C3b to iC3b that inactivates the C3 convertase, and a negative feedback loop involving complement receptor 1 (CR1). These delicately control the spontaneous and immune response‐induced activation of the complement system, avoiding deleterious overactivation. See Figure 2.

### 3.2 Role of complement in AD brain

In a healthy brain, the BBB regulates the influx of invading pathogens, immune cells, and complement from the periphery (Veerhuis, Nielsen, & Tenner, 2011). Resident cells, namely, astrocytes, neurons, and microglia, produce and release complement proteins that may play a role in the maintenance of neural circuitry and regulation of synaptic pruning (Schafer et al., 2012).

Several complement proteins are up‐regulated in human AD (Terai, Walker, McGeer, & McGeer, 1997). Human data and mouse models of AD have indicated the involvement and activation of complement in the diseased brain (Iqbal et al., 2013; LaFerla & Green, 2012). Interestingly, depleting microglia in mouse models of amyloidosis was shown to be neuroprotective, reducing synapse loss and behavioural defects (Chung, Welsh, Barres, & Stevens, 2015; Schafer et al., 2012; Stevens et al., 2007), which has brought about interest in understanding the possible role of complement in microglia function and AD pathology. Specific patterns of complement activation have been observed throughout the different stages of AD progression. In early stages of disease, C1q, C4d, and C3d were found closely associated with Aβ plaques, whereas in later stages, C1q, C4d, and C3d colocalised with neuritic plaques and neurofibrillary tangles. Paradoxically, terminal complement components, such as the MAC, were down‐regulated (Veerhuis et al., 2011).

Microglia have been shown to be directly involved in pruning synapses, modifying synaptic connections via the classical complement cascade in early development, and possibly also in neurodegenerative disease (Crehan et al., 2012). Microglia are the main source of C1q in the CNS and highly express complement receptor 3. C1q is expressed in the adult brain, and protein levels rise with age. C1q binding to Aβ can trigger activation of the classical complement cascade (H. Jiang, Burdick, Glabe, Cotman, & Tenner, 1994). Studies have now shown C1q to be active downstream of Aβ processing. It has also been shown that C1q binds to neuronal presynaptic terminals and triggers local apoptosis (Gyorffy et al., 2018). In response to complement activation, microglia further express receptors for C3a and C5a, in turn triggering inflammation. The tagging of synapses with C1q, followed by opsonisation of synapses by C3b, and the subsequent phagocytosis of the opsonised synapses by microglia has been reported in AD mouse models (Schafer et al., 2012; Stevens et al., 2007). The phagocytosis of synaptic material by microglia was confirmed by light microscopy, which was attenuated in C3 or complement receptor 3 KO mice (Schafer et al., 2012). The engulfment of synapses is hypothesised to vary with different developmental stages, regions of the brain, and disease states (Galatro et al., 2017). Thus, complement plays an important role in maintaining healthy neural plasticity in the early developing brain and additionally may play a role in maintaining the activated disease‐associated state of microglia, especially with relation to phagocytosis of synapses in the diseased adult brain.

Astrocytes play a critical role in the maintenance of CNS homeostasis, by regulating neurological function, synapse formation, and elimination. Chronically activated disease‐associated microglia are present in the CNS tissue of patients with various neurodegenerative diseases, including AD, and they release pro‐inflammatory cytokines, particularly IL‐1α, TNF‐α, and C1q, which lead to downstream activation of astrocytes (Liddelow et al., 2017). Disease‐associated “A1” astrocytes express various complements including C1q, C1r, C1s, C3, and C4 (Stephan, Barres, & Stevens, 2012; Stevens et al., 2007) and down‐regulate various complement regulatory proteins such as CD59, CD46, DAF, and CR1 (Goetzl, Schwartz, Abner, Jicha, & Kapogiannis, 2018). “A1” astrocytes have been identified in several neurodegenerative diseases including AD, Huntington's disease, Parkinson's disease, amyotrophic lateral sclerosis, and multiple sclerosis. C3 was shown to be highly up‐regulated in “A1” astrocytes, wherein nearly 60% of glial fibrillary acidic protein‐positive astrocytes in the prefrontal cortex in human AD were C3 positive suggesting that an integral role of these microglia‐activated. “A1” disease‐associated. astrocytes in disease initiation and progression (Liddelow et al., 2017). Interestingly, neurons respond aberrantly when exposed to astrocytic complement components. In response to Aβ, astrocytes secrete C3 and neurons react by shrinking some synapses and ramping up the activity of others. This response has been associated with NF‐κB signalling (Lian & Zheng, 2016).

Neurons have also shown up‐regulation of various complement proteins in the AD brain (Veerhuis et al., 1998). Neurons express the C3a receptor and respond to NF‐κB and C3 up‐regulation through this receptor. C3 KO, which in theory should block all three activated complement pathways, has shown mixed results in different studies. C3 KO was shown to be neuroprotective in APP/PS1 transgenic mice (Hong et al., 2016; Shi et al., 2017; Shi et al., 2015) and neurodegenerative in other APP transgenic AD mouse models (Maier et al., 2008; Wyss‐Coray et al., 2002). C3 KO has also been shown to be associated with increased Aβ burden (Maier et al., 2008; Shi et al., 2017; Wyss‐Coray et al., 2002). Considering the different phenotypes observed, probably due to the different mouse models employed, further functional studies seem to be required to explore the role of complement in the AD brain.

Due to the discrepancies in the role of complement observed in the different mouse models, great care needs to be taken when translating these findings to humans. Large differences were observed on comparing the expression profiles between mouse and human brain tissues. Complement C2 and C3 genes are highly expressed and regulated in an age‐dependent manner in human microglia, which is not seen in mouse microglia. Aged human microglia showed a pronounced inflammatory response, existing in a preactivated state, which was not the case in the mouse microglia (Galatro et al., 2017; Zrzavy et al., 2017).

### 3.3 Genetic link of complement in AD

Considering the differences in expression observed in mouse and human cells, interest in the field has shifted to focusing on genetic data from human AD patients, in order to elucidate the role of the most important genes. Recent GWAS have linked several complement components to AD risk, specifically CR1, clusterin (CLU; ApoJ), and genes encoding C1s and C9 (Lambert et al., 2009). CR1 is a cell surface receptor for the complement fragments C3b and C4b. It has roles in phagocytosis, immune clearance, and inhibition of the complement cascade (Khera & Das, 2009). CR1 is involved in both classical and alternative pathways, by binding to C3b, C4b, C1q, and mannan‐binding lectin (Crehan et al., 2012). There have been conflicting reports on the expression of CR1 in the human brain. Some studies have shown expression in astrocytes but not in neurons and microglia by immunocytochemistry (Fonseca et al., 2016; Gasque, Dean, McGreal, VanBeek, & Morgan, 2000). Another study showed expression in neurons but not in astrocytes and microglia in the AD brain by Western blotting and immunohistochemistry (Hazrati et al., 2012). Other studies failed to detect any CR1 protein in any of the cell types in the human brain (Johansson et al., 2018). Several coding and non‐coding SNPs have been associated with AD disease incidence and progression, although these vary with populations (Zhu et al., 2015). Two SNPs, rs6656401 and rs3818361, are significantly linked to LOAD in European, American, and Chinese populations. CR1 has four different alleles, each of which produces a CR1 protein varying in size (Crehan et al., 2012). The longer “S” isoform, which contains an extra C3b/C4b binding site, has been shown to be associated with an increased risk of developing AD (Mahmoudi et al., 2015). Unlike in the brain, in the periphery, CR1 is known to play several critical roles such as clearance of complement‐opsonised pathogens by erythrocytes and macrophages through CR1. Recently, studies have shown the interaction of peripheral CR1, namely, through immune adherence via red blood cells with clearance of Aβ (Johansson et al., 2018). Deficits in CR1‐dependent Aβ clearance mechanisms in AD in peripheral erythrocytes and macrophages were reported. Furthermore, it was also suggested that CR1 polymorphisms that decrease CR1 and in turn Aβ clearance increase AD risk and vice versa. This might affect brain Aβ metabolism by impairing efflux of brain Aβ or enhancing the influx of circulating Aβ.

CLU is the third most highly associated risk gene for LOAD with SNP rs11136000 at an odds ratio of 0.840 as detected by GWAS (Lambert et al., 2009). Although not a complement protein itself, it plays a major role as a complement regulator. CLU is a potent regulator of the formation of the terminal complement component, MAC. It was first suggested to have a role in AD when it was observed to be increased in post‐mortem hippocampi of AD patients (May et al., 1990). In rodent models, the KO of the CLU gene led to reduction of fibrillary Aβ, but not total Aβ (DeMattos et al., 2002), and protected rodent neurons from Aβ‐induced cell death (Killick et al., 2014).

### 3.4 Complement‐based therapies: Points of intervention

A range of complement targets have been evaluated in order to modulate complement in disease conditions (Ricklin, Barratt‐Due, & Mollnes, 2017). Approaches include modulation of peripheral targets, inflammatory targets, and central components of the complement system.

One strategy is to block the activation cascade by using inhibitors upstream in the complement system. A preparation of a C1‐inhibitor, Cinryze®, one of the few complement‐associated therapeutic agents to be clinically approved, is used in hereditary angioedema (Zeerleder, 2011). This blocks both the classical and lectin pathways. Annexon is developing a monoclonal antibody that blocks initiation of the complement cascade by acting on C1q, specifically for neurodegenerative purposes (Chakradhar, 2016). Another potential point of intervention is inhibition of the C3 convertase, the point where all the complement pathways converge, which would block both classical and alternative pathways. The greatest consideration when employing strategies where the initiation of one complement pathway is targeted is the effects these inhibitors may have on the other two pathways. Careful target and pathway validation will be paramount to avoid unwanted side effects, given the complexity of the complement system.

Another point of intervention being considered is the control of anaphylatoxins, which are directly involved in the inflammatory response. Approaches include either blocking the receptor, such as C5aR, or directly blocking C5a. A low MW agonist, PMX205 (Alsonex), that targets C5aR1 has shown efficacy in clinical trials and BBB penetration (Hawksworth, Li, Coulthard, Wolvetang, & Woodruff, 2017). A second therapeutic agent licensed for clinical use, eculizumab, is a C5 blocking monoclonal antibody that blocks the formation of the MAC and the production of C5a anaphylatoxin. Although trials employing therapeutic antibodies are ongoing, BBB penetration in general is still one of the biggest hindrances to the application of many therapeutic agents in neurodegenerative diseases.

Inhibitors targeting factor B and factor D, alternative pathway modulators of C3b, would prevent assembly of the C3 convertase. This would have the benefit of not completely inhibiting the complement cascade but would rather control the amplification of the cascade. Genentech has developed lampalizumab, an antifactor D antibody evaluated for age‐related macular degeneration (Katschke et al., 2012). Several low MW inhibitors of factor D have also been developed, but no clinical trials have yet been run for age‐related macular degeneration. Properdin (complement factor B) is an interesting target, functioning as a modulator of C3 convertase in the alternative pathway, by enhancing C3b degradation, or decay of the convertase complex. Several antibodies and small molecules are being developed (Blatt, Pathan, & Ferreira, 2016). Another approach could include targeting C3 directly, by depleting circulating C3 before it is activated. The compstatin family of C3 inhibitors have been shown to protect C3 from binding to the convertases, keeping it inactive (Mastellos et al., 2015). Mirococept, a truncated membrane‐targeted version of human complement receptor 1 (CR1), is currently in trials for kidney transplantation. It consists of three domains, one that contains the biological activities of CR1 and two domains that permit binding and insertion into cell surfaces inhibiting C3 (Smith, 2002).

To summarise, the complement system is a complex first line of defence against pathogens, consisting of more than 15 proteins. In AD, the complement system can function in both a beneficial and detrimental manner. In early stages of the disease, this system may play a protective role in attenuating Aβ pathogenesis, by activating microglia. At the later stages, the complement system may contribute to synapse loss, activation of astrocytes, and release of various inflammatory‐associated cytokines. Evidence suggests an important role of dysregulated complement in AD. Yet, there is still the need to confirm cell type‐specific expression of the different complement factors in the CNS and expression changes in AD. In recent years, we have gained a better understanding of the disease mechanisms and developed new powerful tools to study the different cell types shown to be involved in disease aetiology, in vitro. However, there is still a need for stringent functional studies in human models to facilitate the development of an effective therapeutic strategy. A large number of complement‐targeting therapeutic agents have been developed for other diseases, but their application to AD has been limited. There is a large market and potential in targeting the complement cascade with relation to therapeutic benefits in AD. An interesting point of intervention to consider is the alternative pathway, as blocking this pathway would subtly modulate the activity of the complement system, without disrupting the homeostatic role of the complement cascade.

## 4 TREM2

TREM2 is a gene encoding an IgG‐superfamily immune receptor, with an IgG V‐like region in the extracellular domain, a single‐span transmembrane region, and a short cytoplasmic domain. Several TREM2 mutations have been described in AD, bringing TREM2 to the forefront of neurodegeneration. In this section, we describe TREM2 expression and the role of TREM2 on microglial functions. The effect that neurodegenerative disease‐linked TREM2 variants have on TREM2 expression and function is discussed, with a particular focus on R47H, one of the best characterised AD‐linked variants. Comparisons are made between the phenotype of R47H TREM2 in human AD brain and rodent models of AD with the same mutation or with TREM2 deficiency. Finally, the therapeutic potential of TREM2 and its downstream signalling pathway is considered.

### 4.1 TREM2 expression and function

TREM2 was first cloned in 2000, following a human cDNA library screen for novel immune‐activating receptors related to NKp44, an activating NK cell receptor. Expression of TREM2 was noted at the time to be present on macrophages and dendritic cells but not monocytes or granulocytes (Bouchon, Dietrich, & Colonna, 2000). In the brain, TREM2 is expressed in microglia and perivascular macrophages but not in neurons and other brain cell types (Kober & Brett, 2017). Protein expression of TREM2 is low in resting microglia but is highly up‐regulated in microglia surrounding amyloid plaques in AD brains and also appears to be high in infiltrating macrophages at early cerebral infarcts (Fahrenhold et al., 2017; Lue et al., 2015).

**Figure 3:**
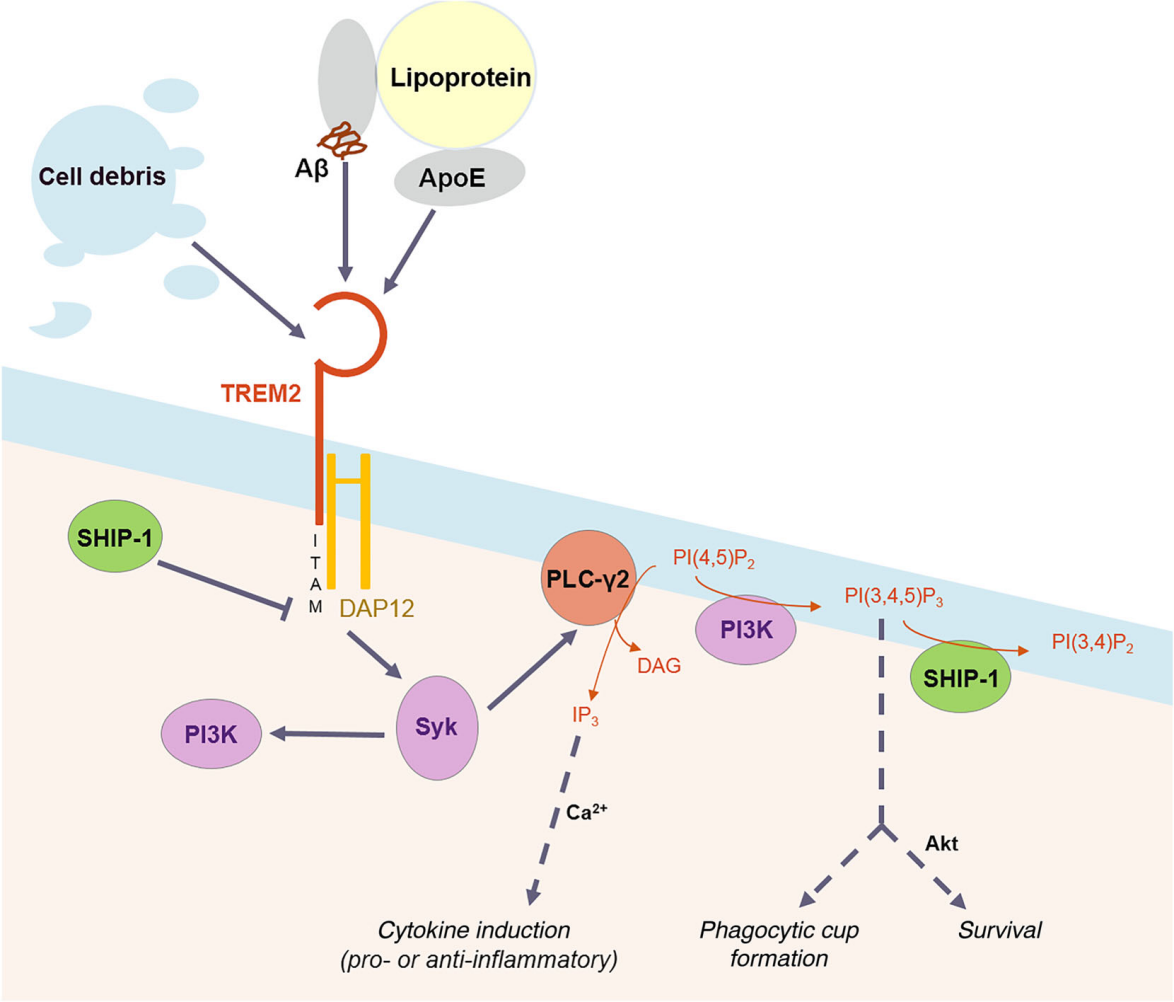
TREM2 signalling. The TREM2 receptor is activated by many ligands including anionic lipids on cell debris, Aβ, ApoE, and lipoproteins. TREM2 signals through its ITAM‐domain‐containing co‐receptor DNAX‐activation protein 12 (DAP12), leading to Syk activation. Syk activates PI3K, leading to actin assembly and phagocytosis, and Akt recruitment that triggers pro‐survival signalling. Syk additionally activates PLC‐γ, causing calcium mobilisation and cytokine induction. SHIP‐1 negatively regulates DAP12 and PI(3,4,5)P_3_

TREM2 signals by complexing with a coreceptor called DNAX‐activation protein 12 (DAP12), the ensuing signalling cascade has been characterised only in dendritic cells (Kober & Brett, 2017). Activation of the TREM2‐DAP12 signalling complex induces phosphorylation of the DAP12 immunoreceptor tyrosine‐based activation motifs, and these recruit and activate the kinase Syk, which activates PLC‐γ and PI3K, the latter of which increases production of phosphoinositol (3,4,5) trisphosphate (PIP_3_). Downstream signalling stimulates calcium mobilisation, actin remodelling and the Akt pathway. TREM2‐DAP12 activation regulates survival, proliferation, migration/chemotaxis, and phagocytosis (Kober & Brett, 2017; Takahashi, Rochford, & Neumann, 2005). SH‐2 containing inositol 5′ polyphosphatase‐1 (SHIP‐1; also known as INPP5D) encodes an inositol phosphatase that binds directly to activated DAP12, inactivating the TREM2‐DAP12 signalling complex by preventing further kinase recruitment (Peng et al., 2010). SHIP‐1 dephosphorylates PIP_3_, inhibiting this downstream mediator of DAP12 signalling. See Figure 3.

Tonic TREM2 signalling appears to be necessary to maintain microglial metabolism, via mammalian target of rapamycin activation. In fact, TREM2‐deficient microglia have defective survival, migration, and phagocytosis (Ulland et al., 2017). Furthermore, weak stimulation of TREM2 is anti‐inflammatory and can suppress TLR4‐induced pro‐inflammatory cytokine production, via a poorly characterised mechanism regulated by JNK (Hamerman et al., 2006; Zhong et al., 2015; Zhong, Zhang, et al., 2017). Aside from its role as a membrane receptor, TREM2 undergoes regulated intramembrane proteolysis, with the entire ectodomain shed by the disintegrin and metalloproteinase ADAM 10 or ADAM17 metalloproteinases, depending on the cell model used (Feuerbach et al., 2017; Thornton et al., 2017). The secreted ectodomain, soluble TREM2 (sTREM2), appears to have its own distinct biological activity (Kober & Brett, 2017; Zhong, Chen, et al., 2017). The function of sTREM2 is poorly defined, but it has a pro‐inflammatory effect when injected into mouse brain, and sTREM2 is elevated in the CSF of preclinical AD patients, probably because of microglial activation (Suárez‐Calvet et al., 2016; Zhong, Chen, et al., 2017).

Receptor ligands can also shed some light on receptor function. In the case of TREM2, the ligands uncovered by in vitro studies are incredibly diverse: anionic lipids (e.g., phosphatidylserine), sulphated proteoglycans, anionic bacterial carbohydrates (or whole bacteria), lipidated apolipoprotein E/J, and lipoproteins (Kober & Brett, 2017). TREM2 also binds to oligomeric Aβ, which results in a TREM2‐dependent phenotype in mouse microglia, including increased migration, proliferation, Aβ clearance, and secretion of cytokines IL‐6 and the chemokine CCL3 (Zhao et al., 2018; Zhong et al., 2018). Another ligand relevant to AD is phosphatidylserine, exposed by both transiently stressed and apoptotic neurons (Brown & Neher, 2014). TREM2 overexpression in microglia was shown to promote phagocytosis of co‐cultured apoptotic neurons and to reduce inflammation. In the same study, TREM2 knock‐down inhibited phagocytosis and increased transcription of TNF‐α and inducible NOS (Takahashi et al., 2005). A better understanding of the role of TREM2 in phagocytosing particular ligands is critical, as promoting phagocytic clearance of Aβ and apoptotic cells in AD, while avoiding engulfment of viable stressed neurons, could represent a beneficial therapeutic strategy.

### 4.2 TREM2 loss‐of‐function mutations are associated with neurodegenerative disease

In 2013, rare variants of the TREM2 gene were discovered to be significantly enriched in LOAD patients (Guerreiro et al., 2013; Jonsson et al., 2013). Of these, the R47H (rs75932628) SNP has the strongest association with AD in heterozygous carriers, with an odds ratio of 2.9 in an Icelandic population, although the effect size is modest. Other mutations in TREM2 and DAP12, expressed homozygously, have been described in patients with “Nasu‐Hakola” disease, an autosomal recessive disease that presents with early‐onset dementia and bone cysts (Paloneva et al., 2001). Fronto‐temporal dementia and AD have also been associated with heterozygous expression of “Nasu‐Hakola” TREM2 mutations Q33X and T66M (Borroni et al., 2014). Nasu‐Hakola mutations in TREM2 cause a severe loss of function, with no functional TREM2 protein expressed at the cell surface, compounded by endoplasmic reticulum stress from TREM2 misfolding (Kober & Brett, 2017). In contrast, the AD mutation R47H has a subtle effect on TREM2 cell surface expression, with a slight reduction reported in the BV‐2 microglial cell line (Kleinberger et al., 2014), but no effect in mouse bone marrow‐derived macrophages, T cells, and HEK293 (Cheng et al., 2018; Kleinberger et al., 2014; Wang et al., 2015). The inconsistent surface expression displayed by R47H TREM2 cells in culture may be partly due to its overexpression in cell lines with no endogenous TREM2, which may lack factors that regulate TREM2 trafficking, stability, and signalling. The R47H mutation is situated within the ligand‐binding domain of the protein, and the substitution of arginine for histidine reduces positive charge of the domain (Kober et al., 2016). A recently published crystal structure of the TREM2 Ig‐like domain, with an R47H mutation, has shown that the ligand‐binding CDR2 loop is destabilised and swings out at acidic pH (Sudom et al., 2018). The wild‐type protein structure was crystallised with phosphatidylserine and showed that the Ig‐like domain has multiple ligand‐binding sites that are disrupted by the R47H mutation, which explains its universal reduction in binding affinity (Sudom et al., 2018). Reduced solubility, increased turnover, altered glycosylation, and reduced sTREM2 shedding of the R47H variant had previously been reported in cell culture and in vivo. Thus, the new structural data can shed some light on how these are connected (Park et al., 2015; Park, Ji, Kim, An, & Yoon, 2017; Sudom et al., 2018).

Similar mechanisms are likely to underlie another TREM2 point mutation associated with increased AD risk: R62H, which occurs at the ligand‐binding region in proximity to R47 (Jin et al., 2014; Sims et al., 2017). Another described AD risk variant, H157Y, is notable for increasing sTREM2 shedding in HEK293 cells (T. Jiang et al., 2016; Schlepckow et al., 2017; Thornton et al., 2017). This effect on sTREM2 production is distinct from the R47H and R62H mutations. H157Y TREM2 is predicted to have a functional ligand‐binding domain, unlike R47H and R62H (Schlepckow et al., 2017). The H157Y mutation is within the shedding site and appears to promote ADAM10‐independent shedding (Thornton et al., 2017). Importantly, the increased rate of shedding reduces TREM2 cell surface expression and reduces phagocytosis of Escherichia coli (Schlepckow et al., 2017). Thus far, the common denominator between the AD‐linked TREM2 mutations seems to be a reduction in functional TREM2 receptor signalling.

### 4.3 AD human data and rodent models support a neuroprotective role for TREM2

TREM2 protein levels are increased in human AD cortex (Lue et al., 2015). Microglia highly expressing TREM2 are enriched at amyloid plaques and are frequently found in proximity to neurites with intracellular Tau pathology (Lue et al., 2015). As previously discussed, TREM2 AD‐linked mutations imply reduced TREM2 function. This appears to be incompatible with the human AD expression data. One potential explanation is that TREM2 signalling is a beneficial response that supports microglia activation and migration towards amyloid plaques, helping to prevent amyloid spread and damage. In AD brains with the R47H/R62H mutations of TREM2, fewer microglia associate with amyloid plaques than AD noncarriers, and their processes appear to physically engage less with the amyloid fibrils (Krasemann et al., 2017). Amyloid plaques surrounded by microglia are more compacted, in comparison to diffuse plaques surrounded by fewer microglial cells, and this seems to correlate with decreased neurite degeneration of surrounding neurons (Wang et al., 2016).

Researchers have looked to rodent models to determine whether TREM2 activity is beneficial or detrimental to AD progression. There are three types of TREM2‐centric mouse models that have been crossed with mouse models of AD, to study disease pathology: TREM2 KO, R47H knock‐in TREM2, and overexpressed common variant TREM2.

Several studies of TREM2 KO mice crossed into 5xFAD and APP/PS1 models of AD show a reduction in microglia clustering around plaques, which correlates with observations in human AD post‐mortem tissue. However, the effects on amyloid plaque number in these models vary, depending on the genetic background, age, and brain region examined (Jay, Hirsch, et al., 2017; Jay et al., 2015; Krasemann et al., 2017; Wang et al., 2015; Wang et al., 2016). For example, hippocampi from 8‐month‐old TREM2 KO‐5xFAD mice have more amyloid plaques than 5xFAD mice, whereas at an earlier age of 4 months, there is no difference in hippocampal plaque number (Wang et al., 2015; Wang et al., 2016). The morphology of the plaques, rather than the number, appears to be more indicative of the TREM2 genotype: TREM2 KO‐AD mice present diffused amyloid plaques, with amyloid filaments extending outwards, and fewer compact amyloid plaques than AD mice (Jay, Hirsch, et al., 2017; Wang et al., 2016). In another study, haploinsufficiency of TREM2 (TREM2^−/+^) in AD mice resulted in diminished physical coverage of plaque surface by microglial processes and strongly impaired Aβ phagocytosis (Yuan et al., 2016). The effect on Aβ phagocytosis was not replicated by other studies with TREM2^−/+^‐ and TREM2 KO‐AD mice (Song et al., 2018; Ulrich et al., 2014) and TREM2 KO non‐AD mice (Zhao et al., 2018), reflecting the previously observed inconsistencies in plaque numbers between mouse models.

Pure Tauopathy models have also shown conflicting results when TREM2 was knocked out. TREM2 KO‐hTau mice were shown by one group to have increased pathology and neurodegeneration (Bemiller et al., 2017), while another group reported decreased neuroinflammation and decreased neurodegeneration in the PS19 Tau protein transgenic mouse model (Leyns et al., 2017). In a primary microglia–neuron co‐culture, Tau hyperphosphorylation was shown to result from LPS‐induced inflammation, suggesting that inflamed microglia can induce Tau pathology. Interestingly, in this system, overexpression of TREM2 in the microglia ameliorated the effects on neuronal Tau protein, whereas TREM2 knock‐down worsened Tau hyperphosphorylation (T. Jiang et al., 2018).

Microglia isolated from TREM2 R47H mice exhibit subtle phenotypic defects that confirm a reduction in some TREM2‐mediated functions. R47H TREM2 knock‐in mice, with no AD genetic background, are healthy and show no clinical phenotype, yet their primary microglia showed reduced survival under colony‐stimulating factor 1‐limiting conditions, decreased motility, and impaired chemotaxis compared with wild‐type mice (Cheng et al., 2018). Phenotypic defects were similar, but less severe, in R47H TREM2 compared to TREM2 KO primary microglial cells. Similar phenotypic defects were observed in a separate study of AD mice with overexpressed human R47H TREM2. Defective Aβ‐induced microglial proliferation and chemotaxis was observed, compared to AD mice with overexpressed common variant TREM2 (Song et al., 2018). No reduction in amyloid pathology or phagocytosis was detected in the R47H transgenic mice, but this is not unexpected given the variable amyloid changes in TREM2 KO‐AD mice. It remains to be seen what factors determine the variable effects of TREM2 in mouse AD models and whether this describes how the disease evolves over time in human AD.

Conversely, overexpression of TREM2 appears to slow the progress of AD pathology in mouse models. Yang and colleagues introduced a bacterial artificial chromosome containing human TREM2 and portions of surrounding regulatory elements into AD mice (5xFAD and APP/PS1; Lee et al., 2018). Similar to lentivirus‐expressed TREM2 in an AD mouse model, there was less amyloid burden and improved cognitive function, relative to AD mice without the TREM2 transgene (T. Jiang et al., 2014). In addition to presenting fewer amyloid plaques, the plaques in TREM2‐overexpressing AD mice were more compact and less filamentous and opposite to changes seen in TREM2 KO‐AD mice (Lee et al., 2018).

## 5 TREM2 SIGNALLING PATHWAYS AS A THERAPEUTIC TARGET

The majority of evidence from studies of TREM2 AD‐associated mutations, in vitro and in vivo, indicate that defective TREM2‐DAP12 signalling may worsen disease progression. Therefore, in patients with TREM2 AD‐risk alleles, it would be desirable to rescue defective TREM2‐mediated signalling therapeutically. In sporadic AD patients, it seems that TREM2‐dependent gene expression is already up‐regulated in plaque‐associated microglia, yet a further boost to TREM2 activity may prove beneficial in recruiting microglia to plaques. TREM2‐DAP12 signalling can be activated by specific TREM2 antibodies binding to the ligand‐binding domain, which have been used as an experimental tool. Antibody‐mediated activation of TREM2 has been shown to boost survival and restore migration defects of R47H TREM2 primary microglia but not TREM2 KO microglia (Cheng et al., 2018). However, directly stimulating TREM2 is thought to have two‐pronged effects, either anti‐inflammatory with weak/monovalent stimulation or pro‐inflammatory with strong/multivalent stimulation (Kober & Brett, 2017). Providing a cautionary note, Kobayashi, Konishi, Sayo, Takai, and Kiyama (2016) found that intrathecal administration of “agonistic” TREM2 antibodies into healthy mice induced pro‐inflammatory cytokine expression and neuropathic pain. Whether the detrimental effects resulted from an off‐target immune response or an overactivation of TREM2 is not clear but may be a moot point if the technical challenge of engineering antibodies to cross the BBB is not met.

The regulated shedding of TREM2 extracellular domain by ADAM family enzymes is also a potential target, since this regulates the level of functional cell surface TREM2 receptors. Although ADAM10/17 inhibitors might be effective at enhancing cell surface TREM2 (Kleinberger et al., 2014), unwanted side effects would be anticipated due to the ADAMs multitargeting effects. A selective competitive inhibitor of the TREM2 shedding site would be preferable, although it may be a challenge to achieve sufficient selectivity. Additionally, it will be important to define the biological activity of sTREM2, as the consequences of increasing sTREM2 are not fully understood, in particular with relation to microglial survival (Zhong, Chen, et al., 2017).

Instead of targeting TREM2 itself, an alternative strategy would be to target other regulators of the TREM2‐DAP12 signalling pathway. AD‐linked mutations in a number of genes that converge on TREM2‐DAP12 signalling have been reported. An SNP that elevates AD risk has been described through meta‐analysis of GWAS, which has been proposed to increase SHIP‐1 expression (Jansen et al., 2017). Therefore, inhibitors of SHIP‐1 would be expected to prolong TREM2‐DAP12 signalling by reducing negative feedback. SHIP‐1 inhibitors have been tested systemically in obese mice and were shown to increase IL‐4 production and reduce visceral adipose tissue inflammation, which ultimately reversed the metabolic effects of a high‐fat diet (Srivastava et al., 2016). IL‐4 is known to up‐regulate TREM2 expression, so SHIP‐1 inhibition could indirectly promote TREM2 signalling (Malik et al., 2015). Conversely, another study showed that SHIP‐1 negatively regulates TLR4‐mediated signalling; therefore, knock‐down of SHIP‐1 in cell culture could potentiate inflammation in response to bacterial LPS (An et al., 2005). Although commercially available, the effect of SHIP‐1 inhibitors in AD models has not been reported yet.

The gene PLCG2 encodes the PLC‐γ2, which is downstream of DAP12 signalling in osteoclasts and macrophages (Mao, Epple, Uthgenannt, Novack, & Faccio, 2006). The missense variant p.P522R was reported to reduce risk for LOAD (Sims et al., 2017). This variant lies in the sPH domain, which, structurally, is near the TIM‐barrel domain (Bunney et al., 2009). This domain is a part of the auto‐inhibitory component of the PLCG2 gene and therefore, the described mutation might have an effect on enzyme activity. Development of low MW compounds that mimic the effects of the mutation on enzyme activity could be beneficial in AD.

Finally, another possible way to positively modulate the TREM2 pathway could be represented by targeting APOE. APOE is an apolipoprotein and a ligand of TREM2. KO of APOE in mice prevented the activation of TREM2‐dependent genes (Krasemann et al., 2017). Although SNPs that generate the ε4 variant of APOE have the greatest effect size on AD risk from GWAS, the relationship between APOE‐ε4 and TREM2 activity has not been investigated yet. All of the AD‐linked genes mentioned above that are connected to TREM2 signalling could be further studied as potential therapeutic targets, as they may be more tractable or provide subtler modulation of TREM2‐DAP12 signalling, than targeting TREM2 directly.

In conclusion, TREM2 is a microglial receptor that broadly signals to promote microglial metabolic fitness and survival. This allows microglia to continue performing their brain homeostasis functions, for example, phagocytosis, during periods of increased misfolded protein burden, such as in neurodegeneration. Human genetics clearly links altered TREM2 function to AD. The exact role of TREM2 in AD is still poorly understood, but it is highly up‐regulated in plaque‐associated microglia, and TREM2 deficiency prevents migration of microglia to amyloid plaques, which suggests that TREM2 is necessary for that behaviour. Plaque‐associated microglia express markers of phagocytosis and seem to corral and compact amyloid aggregates, preventing neurite degeneration. Therefore, TREM2 appears to be involved in a beneficial anti‐amyloid response by microglia in AD, which could be useful to exploit therapeutically. Although TREM2 itself is not a conventional “druggable” target, further research into its network of interactors could yield more tractable therapeutic targets.

## 6 CONCLUSION

Microglia are important for regulating homeostasis in the healthy brain, but their exact role in AD has not been settled. AD brains show signs of microgliosis, microglia with altered morphology, clustering around amyloid plaques and dystrophic neurites. This snapshot gives no indication of whether microglia are neuroprotective or neurotoxic and this has to be inferred from human genetics and rodent models. Human genetic evidence, for example, loss‐of‐function mutations in TREM2 associated with increased AD risk, suggests that microglia are neuroprotective. However, many mouse models of AD have shown that microglia depletion reduces AD pathology and neuron death, highlighting the negative role that microglia can have in AD. A likely explanation for these two disparate findings is that microglia restrict accumulation and spreading of protein aggregates early in AD, but once accumulation outstrips clearance, the microglia are driven towards a chronic inflammatory state, in response to cues such as excessive neuron death (Hansen et al., 2018).

Microglia are capable of phagocytosing extracellular Aβ. There are a large number of factors that can contribute to the extracellular build‐up of Aβ aggregates in AD brains, one of which may be reduced microglial phagocytosis, which is observed in rodent models (Krabbe et al., 2013). Oligomeric Aβ is recognised by several microglial receptors, including TREM2. Indeed, TREM2 preferentially binds oligomeric over monomeric Aβ_1–42_. TREM2 mediates both migration towards oligomeric Aβ, clearance of Aβ, and cytokine release in response to oligomeric Aβ (Zhao et al., 2018; Zhong et al., 2018). Whether TREM2 also binds fibrillary Aβ is not yet known. Impaired TREM2 function may therefore affect both phagocytosis and inflammatory responses to Aβ. Microglia in AD are also faced with phagocytosing dead and apoptotic neurons, which are recognised by TREM2 and numerous scavenger receptors. The complement system may also play a role in phagocytosis, allowing dysfunctional synapses to be targeted for removal (Schafer et al., 2012; Stevens et al., 2007).

Neuroinflammation is an important feature of AD, and this seems to be driven by microglia. The real origin of neuroinflammation in AD is not clear, but one mechanism that initiates microglial inflammation in vitro might be the phagocytosis of insoluble protein aggregates, such as Aβ fibrils or oligomers. Aβ both primes and activates the NLRP3 inflammasome, leading to release of the damaging inflammatory mediator IL‐1β (Halle et al., 2008; Stewart et al., 2010). High levels of IL‐1β, TNF‐α, and other pro‐inflammatory cytokines and chemokines are found in the brains and CSF of AD patients (Heneka, Kummer, & Latz, 2014). Neuroinflammation may even be an early feature of AD, since a PET ligand for neuroinflammation has helped to predict conversion from mild cognitive impairment to clinical AD (Cagnin et al., 2001). Complement proteins can also act as pro‐inflammatory mediators secreted by both microglia and astrocytes (Stephan et al., 2012; Stevens et al., 2007). Due to the dual role of complement in both phagocytosis and inflammation, the increased production of C1q and C3 in AD has been proposed to cause inappropriate tagging of healthy synapses for removal, that is, “synaptic pruning.” This process has not been fully delineated in adult neurodegenerative disease brains, but a strong link has been confirmed between C1q/C3 and synapse loss in AD mice (Hong et al., 2016). The cleavage of C3 can also induce the release of TNF‐α, which can in turn lead to activation of downstream mediators of inflammation such as IL‐1, IL‐6, and IL‐18, thus amplifying the inflammatory response (Gasque et al., 2000). Understanding the role that complement proteins play in relation to other known AD‐associated genes, such as the variants of APOE, TREM2, and the inflammasome, will help uncovering the intricate networks that underlie neurodegeneration.

Ultimately, neuroinflammation may worsen the spread of amyloid and Tau pathology, promoting a positive feedback loop that potentiates both neuroinflammation and the accumulation of misfolded proteins. Microglia depletion in mice confers resistance to amyloid‐induced synapse loss (Chung et al., 2015; Schafer et al., 2012; Stevens et al., 2007).

In terms of therapeutic intervention for AD, it seems likely that it might be necessary to intercept more than one target. It has been hypothesised that AD progresses into a “cellular phase” driven by vicious cycles of neuroinflammation (De Strooper & Karran, 2016). To slow the progress of AD, it might be necessary to target amyloid and Tau protein, but also dampen excessive neuroinflammation, and promote healthy microglial surveillance functions in the brain. The human genetics revolution has identified new candidate risk genes for AD in recent years, and researchers are starting to map the common pathways that connect them and characterise their function. We propose that targeting NLRP3 inflammasome activation, complement production and signalling, and TREM2‐DAP12 signalling might provide useful targets for drug development (see Table 1).

**Table 1:** Potential therapeutic targets for AD

Pathway	Target	Justification	Reference
NLRP3 inflammasome	VRAC	Fenamates inhibit VRAC channels which, results in inhibition of NLRP3 inflammasome	Daniels et al., 2016
	P2X7	Activation of P2X7 receptors triggers potassium efflux, which induces NLRP3 inflammasome activation	Solle et al., 2001
	NLRP3	Directly inhibiting NLRP3 would prevent activation of the downstream signalling cascade	Swanton et al., 2018
Complement	C1, C1q, C3	Inhibition would block complement cascade activation	Zeerleder, 2011 Chakradhar, 2016 Mastellos et al., 2015
	CR1	Increase in CR1 would block complement cascade activation	Smith, 2002
	Factor B, Factor D, Properdin	Alternative pathway inhibitors would subtly modulate complement pathway activity and inhibit the amplification arm	Katschke et al., 2012 Blatt et al., 2016
TREM2‐DAP12 signalling	TREM2	Activation of TREM2 in R47H TREM2 mice improves myeloid cell survival and migration defects	Cheng et al., 2018
	SHIP‐1	SHIP‐1 inhibits TREM2‐DAP12‐induced signalling	Peng et al., 2010
	PLC‐γ2	Structural data suggest that a point mutation reduces AD risk and may positively modulate enzyme activity	Mao et al., 2006; Sims et al., 2017
	Apolipoprotein E	Activation of TREM2‐dependent genes is prevented in APOE KO mice	Krasemann et al., 2017

*Note*: The table summarises possible therapeutic targets involved in the NLRP3 inflammasome activation, the complement system, and TREM2‐DAP12 signalling pathways. AD: Alzheimer's disease; CR1: complement receptor 1; TREM2: triggering receptor expressed in myeloid cells 2; SHIP‐1: SH‐2 containing inositol 5′ polyphosphatase‐1; DAP12: DNAX‐activation protein 12; KO: knockout.

Currently, the pharmaceutical industry is progressing therapies that target amyloid and Tau protein, which opens the field for enterprising institutes, such as the Oxford Drug Discovery Institute, to explore and de‐risk novel candidate inflammatory risk genes. Development of lead compounds could be pursued within such institutes and advanced with industrial partners, accelerating progress towards a cure for dementia.

### 6.1 Nomenclature of targets and ligands

Key protein targets and ligands in this article are hyperlinked to corresponding entries in http://www.guidetopharmacology.org, the common portal for data from the IUPHAR/BPS Guide to PHARMACOLOGY (Harding et al., 2018), and are permanently archived in the Concise Guide to PHARMACOLOGY 2017/18 (Alexander, Christopoulos et al., 2017; Alexander, Fabbro et al., 2017a: Alexander, Fabbro et al., 2017b; Alexander, Kelly et al., 2017; Alexander, Peters et al., 2017).

## CONFLICTS OF INTEREST

The authors declare no conflicts of interest.
